# Multi-Modal Imaging in Down's Syndrome: Maximizing Utility Through Innovative Neuroimaging Approaches

**DOI:** 10.3389/fneur.2020.629463

**Published:** 2021-01-07

**Authors:** Stephanie S. G. Brown, Elijah Mak, Shahid Zaman

**Affiliations:** ^1^Cambridge Intellectual and Developmental Disabilities Research Group, Department of Psychiatry, University of Cambridge, Cambridge, United Kingdom; ^2^Department of Psychiatry, University of Cambridge, Cambridge, United Kingdom

**Keywords:** Down's syndrome (DS), Alzheimer's disease, multi-modal, MRI, PET, neuroimaging

## Abstract

In recent decades, the field of neuroimaging has experienced a surge of popularity and innovation which has led to significant advancements in the understanding of neurological disease, if not immediate clinical translation. In the case of Down's syndrome, a complex interplay of neurodevelopmental and neurodegenerative processes occur as a result of the trisomy of chromosome 21. The substantial potential impact of improved clinical intervention and the limited research under-taken to date make it a prime candidate for longitudinal neuroimaging-based study. However, as with a multitude of other multifaceted brain-based disorders, singular utilization of lone modality imaging has limited interpretability and applicability. Indeed, a present challenge facing the neuroimaging community as a whole is the methodological integration of multi-modal imaging to enhance clinical understanding. This review therefore aims to assess the current literature in Down's syndrome utilizing a multi-modal approach with regards to improvement upon consideration of a single modality. Additionally, we discuss potential avenues of future research that may effectively combine structural, functional and molecular-based imaging techniques for the significant benefit of the understanding of Down's syndrome pathology.

## Introduction

Down syndrome (DS) is the leading genetic cause of intellectual disability, with an approximate incidence of 1 in 750 live births (1). The extra copy of chromosome 21 is associated with a 4–5-fold overexpression of the amyloid precursor protein (APP) gene and increased accumulation of cerebral beta-amyloid (Aβ) deposition in the brain and subsequent neurofibrillary tau formation, metabolic changes and neurodegeneration (2). The processing of APP generates Aβ, the abnormal accumulation of which leads to the formation of amyloid plaques and the clinical manifestation of early onset (in the forties) Alzheimer's disease (AD) and progressive cognitive decline among individuals with DS. As our understanding of DS has increased and clinical care has improved, the lifespan of individuals with DS in developed countries has improved dramatically (3). However, there are limited and only symptomatic treatments available for AD dementia. Nevertheless, the near ubiquity of AD progression in DS means that multi-modal neuroimaging studies in DS may help delineate the natural history of biomarker changes, and in the process identify early neural substrates of AD pathogenesis. In addition, a multi-modal neuroimaging approach would be especially beneficial due to the genetics of DS and an expected predictable trajectory of development of disease and disorder.

Advances in our understanding in both the some aspects of the molecular basis and the pathogenesis of AD have resulted in novel opportunities to study potential therapeutic targets (4). Neuroimaging contributes to these rising developments by providing biomarkers that could improve comorbid AD diagnosis, inform prognosis, aid in deep phenotyping, allow for risk stratification, and track therapeutic efficacy in future clinical trials for people with DS. Models of AD pathophysiology propose a sequential progression of brain changes that are reflected by neuroimaging abnormalities, beginning with an early increase in Aβ PET tracer binding, followed by a gradual progression of neurofibrillary tau tangles, deficits in cerebral glucose metabolism (i.e., [^18^F]-fluorodeoxyglucose-PET (FDG-PET) and gray matter atrophy and white matter dysfunction as seen with structural T_1_-weighted MRI, resting-state connectivity differences (5) and diffusion imaging (6). Indeed, as we will summarize in the next section, these stereotypical AD biomarker changes have often been recapitulated in the small but growing number of neuroimaging studies in DS.

However, there is still a considerable gap between research findings and translatability into clinical practice for DS. Much progress is still needed to understand the optimal use of these imaging markers and how they could be jointly evaluated during the different stages of the disease. The majority of the literature has hitherto relied on single-modality designs to study pathological processes in isolation, despite the growing appreciation that dementia and DS is a multifactorial disease that likely emerges as a phenotypic spectrum from the dynamics between multiple pathological processes. Further studies that are able to integrate other biomarkers known to play a role in the physiopathology of AD (tau, inflammation, etc.) within a longitudinal design would be useful to unravel their relative roles, sequence, and causal relationships in the context of DS. To this end, large-scale initiatives such has ABC-DS have begun and the research community would be to synthesize the vast amounts of data meaningfully to help us understand better the neuropathological processes and clinical manifestations.

To continue advancement of understanding in the field of Down's syndrome neurodegeneration, it is key that recent innovations in neuroimaging technology are harnessed. As integration of more than one imaging modality leverages significantly more pathology-relevant information that standard methodologies, it is of importance that approaches for doing so are put forward and discussed. In this brief review, we highlight prominent existing research that utilizes such multimodal neuroimaging methods and consider future directions in a methodological context.

## Summary of Literature

### Amyloid and Tau

Post-mortem studies have indicated that virtually all persons with DS over age 40 harbor abnormal degrees of Aβ accumulation (7). This post-mortem evidence has since been borne out by *in vivo* evidence using [^11^C]-PiB PET imaging (8, 9). In addition, several reports have highlighted the striatum as a possible nidus of amyloid accumulation, as it is commonly associated with the earliest and most prominent signal retention (8, 10), similar to that observed in a [^11^C]-PiB PET study of presenilin-1 (PS1) mutation carriers (11). The clinical relevance of Aβ in DS has been highlighted through its associations with brain atrophy (12) and mild cognitive impairment in DS (13). While longitudinal data is scarce, Aβ accumulation in DS rates are similar to that observed in late-onset AD (14), despite an earlier onset by ~15–20 years (8, 10). More recently, tau accumulation in adults with DS has been studied using the PET tracer [^18^F]-AV-1451. The well-documented coupling between tau and amyloid has also led to proposals that elevated tau, particularly its propagation beyond the medial temporal cortices, is predicated upon Aβ positivity (15), consistent with that noted in late onset AD (16). Further studies to investigate the extent to which tau is associated with downstream brain atrophy (17) and cognitive decline is a subject of future research.

### Metabolic Function

Glucose metabolism is one of the most robust biomarkers of AD, and [^18^F]-FDG PET is widely recognized as a sensitive tool to measure neuronal activity on glucose metabolism. In DS, the spatial distribution of hypometabolism appears to resemble that of sporadic AD, encompassing key regions such as the posterior cingulate and other regions that are key nodes of the default mode network (18, 19). Decreased metabolic activity has also been related to elevated Aβ burden and cognitive function (9, 20–23). Recently, the relative tracer delivery (R_1_) from [^11^C]-PiB PET imaging has been validated by Mak et al. (24) as a surrogate index of cerebral perfusion, showing marked deficits that were associated with amyloid deposition and longitudinal cognitive decline.

### Structural MRI

The cortical signature of DS has been a subject of extensive investigations using T_1_-weighted MRI (9, 12, 19, 25). A common finding in the literature points is that of Aβ-associated atrophy in temporo-parietal cortices and subcortical atrophy, hallmarks of atrophy patterns in sporadic AD (26). Interestingly, there are also several reports of increased cortical thickness in DS individuals without amyloid burden (25). Taken together, atrophy appears to be predicated on the presence of amyloid, a notion that is supported by a previous study demonstrating a correlation between Aβ and atrophy in DS (15).

### Diffusion Tensor Imaging

Diffusion tensor imaging (DTI) is a non-invasive *in vivo* technique for characterizing the microstructural properties of white matter tracts by quantifying changes in both the rate and directionality of water molecules (27). However, this topic has received relatively little attention compared to gray matter analyses. One study showed that persons with DS and dementia have decreased white matter integrity compared to non-demented DS subjects in a similar topography seen in AD (28, 29). Given that the role of amyloid and tau accumulation underpinning white matter dysfunction is still unclear, our group is pursuing this topic via a joint-analysis of [^11^C]-PiB, [^18^F]-AV1451 and DTI datasets.

### Methodological Future Directions

The intersection of the clinical understanding of brain pathology and technological innovation in neuroimaging has yielded significant progress in the fields of psychiatry and neurology. However, single modality imaging is inherently limited in the information it can reveal, restricting the understanding of complex and multifaceted disease processes. The complexity problem of neuroimaging datasets in neurological study and the dynamic nature of pathology is difficult to address. The successful and meaningful integration, therefore, of multiple brain imaging modalities is key to clinical progress, whether that be rooted in biomarker identification or translation to therapeutics.

Machine learning is emerging as a significantly useful technique which can both reduce and utilize the high dimensionality of neuroimaging data (30). Thus, supervised machine learning approaches are excellent candidates for the integration of multiple imaging modalities and data types. The basis of machine learning is that a data-driven methodology is applied to predictively model either a discrete or continuous outcome variable. For instance, a well-performing model based on medically non-invasive data, such as structural or functional MRI, which effectively predicts a variable only identifiable to date in an invasive or costly manner, such as amyloid burden in Alzheimer's disease, is of considerable use in clinical practice. In Down's syndrome, such advances are particularly warranted given the need for careful ethical considerations of patient vulnerability and burden. It should be noted however, that unbiased and large datasets are required for such machine learning approaches, as smaller data with sample bias may risk models that are ungeneralizable to the wider population.

Supervised machine learning algorithms are commonly applied using either a classification or regression approach, where the outcome variable of interest is either binary or continuous, respectively (31, 32). These methodologies allow for a tailored hypothesis-driven approach to the data. In Down's syndrome, an important example of the utility of a classifier would be using brain MRI data to define a patient as positive or negative for amyloid burden, thus removing the need for expensive and potentially distressing PET scanning. Given that amyloid positive status is an early indicator of the definitive development of Alzheimer's pathology, this methodology may be utilized as a biomarker for the indication of need for therapeutic intervention. Similarly, the training of regression machine learning algorithms on multimodal brain imaging data may infer accurate predictions of CSF-based biomarkers without the need for invasive procedures and laboratory cost. Importantly, machine learning represents a significantly useful technique for the integration of different types of brain imaging modalities, which among other benefits, such as predictive power, provides a wider and more accurate insight into brain pathology, leveraging multiple data types meaningful to underlying biology (33). Additionally, such implementation of machine learning for multimodal neuroimaging datasets would allow for the assessment of models for predicting pertinent clinical states and, where longitudinal training data is available, prognostic value. The limitations of machine learning techniques however should also be acknowledged, such as to-date minimal uptake of published algorithmic diagnostic approaches into clinical communities and the necessity of large and generalizable datasets for training. DS-AD is a promising candidate for such methodology as on the whole, very high disease penetrance means that the clinical population is relatively homogenous compared to other psychiatric disorders and dementias. To address the issue of translation into the clinic, neuroimaging research employing machine learning tools should carefully consider both the representative nature of the dataset and test hypotheses that address significant clinical need.

Additionally, when considering brain structure and connectivity, statistically integrating multiple MRI techniques can also be advantageous in understanding fundamental biological mechanisms. Two of the most commonly acquired types of structural data are T1-weighted and diffusion-weighted images, which rely on the time taken for the proton spin to realign with the static B_0_ magnetic field and the anisotropy of water diffusion along multiple magnetic gradient b-vectors, respectively (34). Therefore, the combination of T1-weighted and diffusion-weighted imaging allows for tissue macrostructure and the tissue integrity to be analyzed in tandem. One example of such an approach is the using statistical covariance of regional brain structural metrics and diffusivity metrics like fractional anisotropy to produce a morphometry similarity index, which has been previously used to demonstrate significant underlying pathology in disorders such as psychosis (35). Application of such integration between structural modalities gives enhanced understanding of alterations to brain tissue during disease states and would be particularly useful in mapping AD development in Down's syndrome.

**Figure 1:**
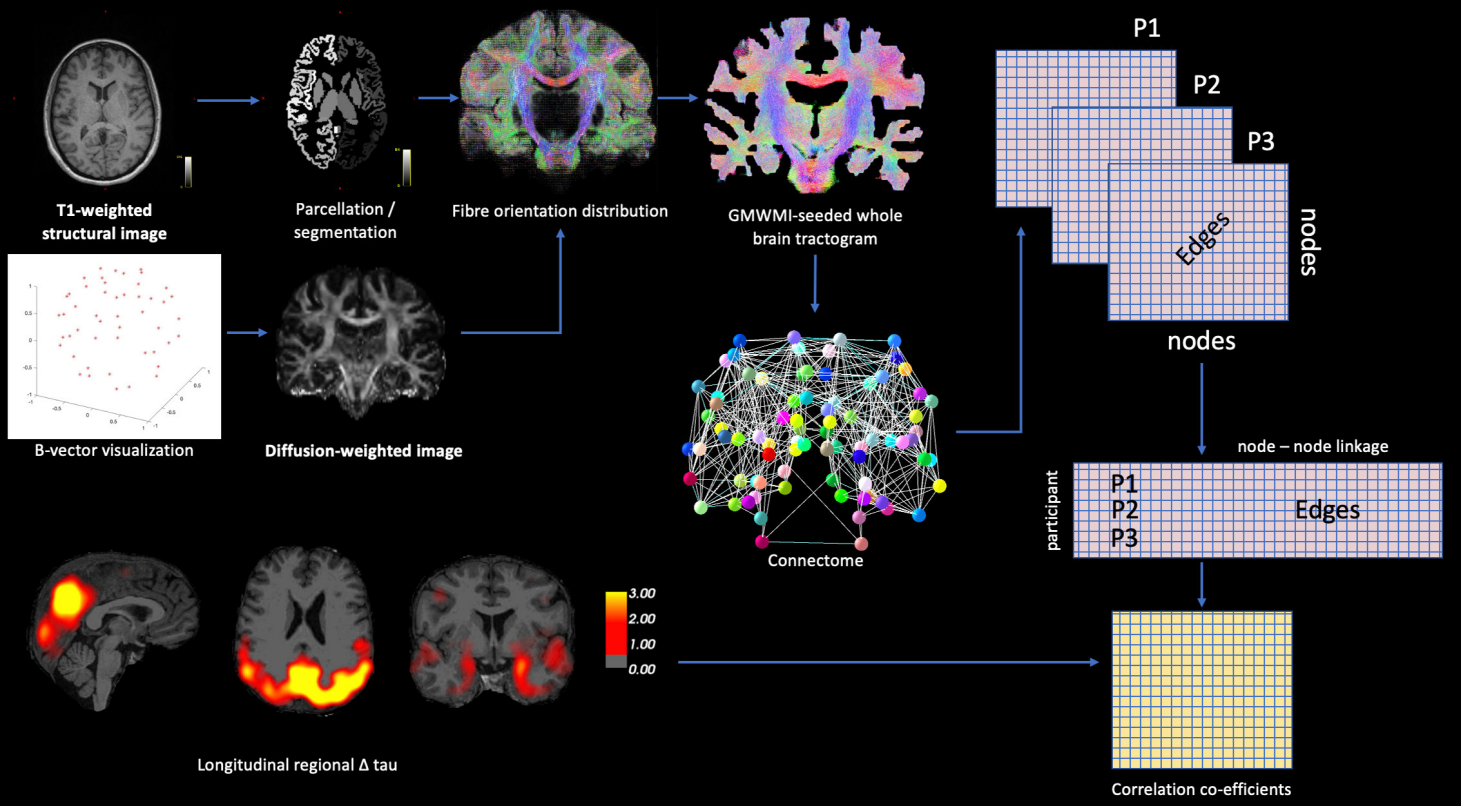
Theoretic example of how connectomic approaches may be integrated with PET-based neuropathology data. Diffusion-based white matter connectivity can be statistically modeled with PET ligand binding using matrix methodology.

When considering PET modalities, atlas-based parcellations of T_1_-weighted structural images are routinely used to label anatomical brain regions for the extraction of degree of tracer binding. However, other modalities may be leveraged in this approach to assess neuropathology in tandem with other metrics, such as functional connectivity, as demonstrated by Franzmeier et al. (36). Through the application of linear regression, with the vectorized functional connectivity matrix serving as a predictor for degree of longitudinal tau change as measured by AV1451, the results showed a functional connectivity and tau-spread coupling, which supports the hypothesis of trans-neuronal tau propagation in sporadic AD. Hence, the multimodal approach to data analysis in this case yielded important results for understanding the pathological development mechanisms of AD. In the case of DS-AD, connectomic approaches may be used in a similar way, in tandem with PET-based neuropathology data, for example to assess tau propagation with respect to white matter structural density. The outline of such a methodological approach is summarized in Figure 1.

The increasingly common usage of ultra-high field MRI in clinical research is also a valuable asset for the advancement of methodologies that integrate multiple brain imaging modalities. The enhanced signal-to-noise ratio and spatial resolution of high field scanners, along with the application of Bayesian probabilistic approaches to structural data has allowed for the automated segmentation of small brain structures with previously unattainable accuracy (37). The hippocampal subfields (dentate gyrus, cornu ammonis 1–4 and subiculum) can now be isolated and volumetrically analyzed from T_1_- and T_2_-weighted acquisitions (38, 39), with the potential to provide valuable insight in Down's syndrome given the prominent role of the hippocampus in Alzheimer's neuropathological development. However, to gain a fuller understanding of how AD development affects the hippocampal subfields, a multimodal approach should be leveraged. The treatment of the subfields as regions of interest for tau or amyloid PET analysis would yield significant insight if utilized cross-sectionally across age groups, enabling trajectory-mapping of AD pathology within the hippocampal structure. Additionally, the integration of the hippocampal subfields with structural or functional brain connectivity data as seed regions of interests would progress past basic whole brain analyses and demonstrate hippocampal-specific changes in connectivity and its relevance to clinical phenotypic expression that may further understanding of the pathological role of these granular regions during the development of DS-AD.

The statistical joint analysis of multi-modal imaging data is also a subject of future interest (40). The recently proposed non-parametric combination (NPC) technique could be employed to investigate how multiple imaging measurements are simultaneously associated with dementia or amyloid burden in DS (i.e., “Is amyloid burden associated with both gray matter and perfusion deficits in the same regions?”). For instance, it has been shown that NPC could yield additional information about patterns of group differences not visible in each modality alone (41). To the best of our knowledge, no study has applied this technique to investigate joint changes in multiple modalities and thus should be an area of future research, particularly in the early stages such as DS persons who are cognitively normal.

## Conclusions

In conclusion, we present in this review the trajectory of innovation regarding the integration of multimodal MRI in the clinical research of DS-AD. Whilst this review is not a comprehensive review of all neuroimaging-based methodologies, importantly, we highlight how broad approaches to neuroimaging should continue to develop how modalities can be combined to improve current knowledge of AD pathological development. A benefit of acquiring multimodal neuroimaging data is that different data types can infer different biological mechanisms—for example regional brain metabolism can be inferred from fluorodeoxyglucose (FDG)-PET, broad neurotransmitter and metabolite levels can be extracted from spectroscopy methods and brain structural analyses can examine how regional volumetrics and form associate with neurocognitive measures. The synergistic advantages of combination of these techniques mean that longitudinal imaging studies are valuable for the understanding of pathology and trajectory of disease. Building the evidence base of the development of neuropathology in DS-AD is crucial therefore, not just for more efficient diagnoses, but for identifying pertinent disease stages for effective intervention, potentially modifiable risks and mechanisms or patterns of brain alteration that can be therapeutically targeted. Gaining such knowledge as the degree of involvement of the noradrenergic and cholinergic systems in DS-AD through regional analyses of specialized brain nuclei is one example of how this may be achieved. In this review, we also put forward potential methodological approaches for future research in the field, with the aims of demonstrating that novel advancements in how data is combined can yield pertinent insights into neuropathology and begin to translate research more effectively into patient benefit.

## Author Contributions

SB and EM contributed equally to manuscript writing and conception. SZ contributed to manuscript conception and provided manuscript feedback. All authors contributed to the article and approved the submitted version.

## Conflict of Interest

The authors declare that the research was conducted in the absence of any commercial or financial relationships that could be construed as a potential conflict of interest.
